# Deciphering the role of tryptophan metabolism-associated genes ECHS1 and ALDH2 in gastric cancer: implications for tumor immunity and personalized therapy

**DOI:** 10.3389/fimmu.2024.1460308

**Published:** 2024-09-12

**Authors:** Lexin Wang, Xue Zhou, Haisheng Yan, Yaping Miao, Binbin Wang, Yuheng Gu, Weining Fan, Ke Xu, Shangke Huang, Jie Liu

**Affiliations:** ^1^ General Hospital of Ningxia Medical University, Department of Clinical Medicine, Yinchuan, Ningxia, China; ^2^ Ningxia Medical University, Department of Clinical Medicine, Yinchuan, Ningxia, China; ^3^ Department of Oncology, The Affiliated Hospital of Southwest Medical University, Luzhou, China; ^4^ Intensive Care Unit, Xichong People’s Hospital, Nanchong, China; ^5^ Department of Oncology, Chongqing General Hospital, Chongqing University, Chongqing, China; ^6^ Department of General Surgery, Dazhou Central Hospital, Dazhou, China

**Keywords:** gastric cancer (GC), tryptophan metabolism-associated genes (TMGs), tumor microenvironment (TME), ALDH2, ECHS1

## Abstract

**Background:**

Tryptophan Metabolism-associated Genes (TMGs), such as ECHS1 and ALDH2, are crucial in cancer progression through immunosuppressive mechanisms, particularly in Gastric Cancer (GC). This study explores their effects on the Tumor Microenvironment (TME). Additionally, it examines their potential as novel immunotherapy targets.

**Methods:**

We utilized single-cell and bulk transcriptomic technologies to analyze the heterogeneity of GC. Non-negative Matrix Factorization (NMF) clustering identified key TMGs, and extensive RNA-seq analyses were performed to pinpoint prognostic genes and potential immunotherapy targets. Furthermore, through PCR analyses we found that ECHS1 and ALDH2 gene expression plays a regulatory role in the migration, invasion and inflammatory factor in AGS and SNU-1 cell lines. The interference effect of si-ECHS1 and ad-ALDH2 was validated using cell scratch assay in AGS and SNU-1 cell line.

**Results:**

We observed a statistically significant correlation between ECHS1 and ALDH2 expression and increased TME heterogeneity. Our findings also revealed that ECHS1 down-regulation and ALDH2 up-regulation contribute to reduced TME heterogeneity, decreased inflammation, and inhibited AGS and SNU-1 tumor cells migration and proliferation. GSVA enrichment analysis highlighted the NF-kappa B(NF-κB) signaling pathway as specifically regulated by TMGs. Furthermore,ECHS1 and ALDH2 modulated CD8+ and CD4+ T cell activities, impacting GC progression. *In vitro* experiments further solidified our conclusions by showcasing the inhibitory effects of Si-ECHS1 and ad-ALDH2 on the invasive and proliferative capabilities of AGS and SNU-1 cells. Moreover, Si-ECHS1 and ad-ALDH2 gene expression effectively reduced the expression of inflammatory factors IL-10,IL-7,CXCL8 and IL-6, leading to a remarkable alleviation of chronic inflammation and the heterogeneous nature of the TME.

**Conclusion:**

This research highlights the importance of ECHS1 and ALDH2 in GC progression and immune modulation, suggesting that targeted therapies focusing on these genes offer promising avenues for personalized immunotherapy in GC. These findings hold potential for improving patient survival and quality of life. Future studies on the NF-κB signaling pathway’s role in this context are warranted to further elucidate the mechanisms underlying TMG-mediated immune modulation in GC.

## 1 Introduction

GC is widely recognized as a major type of cancer that contributes to cancer-related deaths globally, with an increasing incidence each year (1–3). It ranks second in morbidity and mortality rates, trailing only behind lung cancer. Alarmingly, over 80% of patients are diagnosed with an advanced GC during initial treatment (4). Recent research underscores the critical role of understanding the TME in advancing immunotherapy for GC. Additionally, disruptions in the TME have been linked to alterations in immune responses and tryptophan metabolism (5). However, an abnormality in tryptophan metabolism leads to accelerated progression of GC and decreased patient survival (6–9). Hence, elucidating the interplay between tryptophan metabolism and the TME in GC is imperative. Studies published recently have shown that tumor cells can sustain cell proliferation and progression by adapting to the regulation of metabolic patterns to obtain essential nutrients from a nutrient-deficient environment. Furthermore, these cells can modify the tumor immune microenvironment (TIME) (10–13).

Tryptophan metabolism plays a crucial role in tumor cell activities and significantly regulates protein synthesis during cell proliferation. Emerging evidence supports that cancer, neurodegenerative disease, inflammatory bowel disease, and cardiovascular disease are significantly associated with the regulation of tryptophan metabolism (14–17). Recent research has shown that tryptophan metabolism, as an important nutrient *in vivo*, plays a significant role in the development of cancer due to its disorder. This is especially evident in cases of abnormal energy metabolism and nutrient provision (18). In breast cancer, we found that disordered tryptophan metabolism increases the tumor immune microenvironment (19). Additionally, tryptophan metabolism is primarily regulated by three rate-limiting enzymes: kynurenine monooxygenase (KMO), indoleamine 2,3-dioxygenase (IDO), and tryptophan 2,3-dioxygenase (TDO). These key regulatory enzymes offer therapeutic targets for several diseases, including tumors, shedding new light on disease treatment strategies. The metabolism of tryptophan also involves the kynuridine, 5-hydroxytryptamine, and indole pathways (20). This indicates that tryptophan metabolism pathways could potentially be key to inhibiting tumor growth. Studies have highlighted the significant role of tryptophan metabolism-related gene IDO2 in tumor progression and the immune response against tumors by influencing crucial metabolic pathways. However, the specific regulatory role of tryptophan metabolism in GC has not been thoroughly documented, and its role in the development of GC has not been reported.

As the bioinformatics technology constantly updated, it is a new breakthrough to explore the regulatory mechanism of TME in GC. RNA sequencing and single-cell transcriptome technology can well reveal the complex relationship between TME and GC, especially the discovery of cell communication and the regulation of transcription factors. For example, we also found the expression and function of ECHS1 and ALDH2 genes by bioinformatics technology.

The ECHS1 and ALDH2 genes are key members of the tryptophan metabolism family. In most cancers, the expression of ECHS1 is upregulated, including in non–small cell lung cancer, pancreatic cancer, and colon cancer (21–23). The expression of ECHS1 is correlated with lipid metabolism and metastasis. Additionally, we found that decreased expression of ALDH2 plays an important role in the activation of hepatocellular carcinoma carcinogenic pathways (24–26). It is particularly interesting that we have identified a crucial protein, ECHS1 and ALDH2, involved in regulating these genes, which is abnormally expressed in epithelial cells of GC, thereby accelerating epithelial carcinogenesis. However, there is still limited research on the role of TMGs in GC. The regulation of tryptophan metabolism could impact the progression of GC. Our study indicates that TMGs contribute to increasing heterogeneity in the TME of GC and enhance cell proliferation and invasion abilities. Moving forward, our research will focus on treating tryptophan metabolism as a strategy to mitigate the influence of the TME in GC.

## 2 Methods

### 2.1 Analysis of GEO and TCGA database progression

In our study, we targeted GC patients and healthy individuals as research subjects, utilizing data from the TCGA databases (https://www.cancer.gov/ccg/research/genome-sequencing/tcga) and GEO databases (https://www.ncbi.nlm.nih.gov/gds/). Specifically, we have focused on datasets of TCGA-STAD, GSE79973, GSE62254, GSE54129, GSE34942 and GSE5118986 for GC.

### 2.2 scRNA-seq data processing

For single-cell RNA sequencing (scRNA-seq) analysis, we obtained the 10× scRNA-seq dataset GSE163558 from the GEO database. We utilized “Seurat4.0” R package to integrate all samples. Quality control (QC) filters were applied using the following parameters, similar to what has been reported:(1) cells with <200 genes were excluded; and (2) cells with >30% mitochondrial RNA reads were excluded. Following normalization using the “LogNormalize” method, we conducted principal component analysis (PCA) on the top 2500 genes and applied uniform manifold approximation and projection (UMAP) for visualizing cell distribution (27, 28). Cell type identification was performed using specific gene markers. Subsequently, we used the “CellChat”R package for cell-cell communication analysis and network visualization. Subsequently, we identified markers to classify GC cell subsets for future analysis.

### 2.3 NMF classification of GC patients cluster in scRNA-seq

Non-negative matrix factorization (NMF) was carried to divide patients into different subtypes according to the following steps: 1) the univariate Cox regression analysis was performed to identify potential prognostic EDGs (*P*<0.05, logFC>1); 2) Performing sample clustering using the SNMF/R method was suitable for sparse data in scRNA-seq. This can be achieved using the “NMF” package.

### 2.4 Survival analysis

The effect of ECHS1 expression on the prognosis of GC patients was analyzed. This was conducted using the Kaplan-Meier plotter, a tool for the meta-analysis-based validation and discovery of biomarkers correlated with survival.

### 2.5 Identification of genes associated with TMGs

Two machine learning algorithms, Random Forest (RF) and Support Vector Machine Recursive Feature Elimination (SVM-RFE), were used to identify important biomarkers in tryptophan metabolism. The “randomForest” R package in R was used to implement the Random Forest technique. The validation set, used to fully analyze the utility of the identified biomarkers, was obtained from the GSE79973 dataset. Subsequently, the prediction ability of the algorithms was evaluated based on Receiver Operating Characteristic (ROC) curve analysis, and the area under the curve (AUC) was calculated.

### 2.6 Cell culture

AGS and SNU-1 cells, both sourced from Beijing, China, were cultured in a medium containing 10% FBS (fetal bovine serum) from Gibco, USA, at 37°C in a 5% CO2 atmosphere. After, we performed transfections with si-ECHS1 and ad-ALDH2, dividing the cells into distinct groups.

### 2.7 The expression of ALDH2 and ECHS1 by qRT-PCR in AGS and SNU-1 cells

Total RNA was extracted from AGS and SNU-1 cells using an RNA extraction kit provided by Aibotek Biotechnology Company, Wuhan. Subsequently, the mRNA expression levels were determined using primers. (Species of Human Origin) IL7 Forward Primer: TTGGACTTCCTCCCCTGATCC, reverse primer TCGATGCTGACCATTATAACAC; (Species of Human Origin) IL10 Forward Primer: GACTTTAAGGGTTACCTGGGTTG,Reverse Primer: TCACATGCGCCTTGATGTCTG;(Species of Human Origin) IL6 Forward Primer: TAGTCCTTCCTACCCCAATTTCC,Reverse Primer: TTGGTCCTTAGCCACTCCTTC; (Species of Human Origin) ECHS1 Forward Primer: CTGTTACTCCAGCAAGTTCT,Reverse Primer: TCACACATCATGGCAAGCTCA; (Species of Human Origin) CXCL8 Forward Primer: ACTGAGAGTGATTGAGAGTGGAC, Reverse Primer: AACCCTCTGCACCCAGTTTTC;(Species of Human Origin) ALDH2 Forward Primer: GGAATTTCCCGCTCCTGATG,Reverse Primer: CACATAGAGGGCGGTGAGG For reverse transcription of RNA into cDNA, we utilized the RNA reverse transcription kit from TaKaRa, Japan. At last, PCR signals 2^-ΔΔCt^ was used to calculate the expression of genes mRNA levels.

### 2.8 Tryptophan metabolism expression level

Tryptophan metabolism detection Assay following steps: 1) Add a certain dilution of the sample to be tested, 100 μ Incubate at 37°C for 1 hour in the reaction wells already coated; 2) Add fresh diluted calibration sample (diluted according to the instructions) to each reaction well for 100% μ L. Incubate at 37°C for 0.5-1 hour and wash three times; 3) Finally, add substrate solution to each reaction well for color development μ L. 37°C for 10-30 minutes. Within 30 minutes, measure cells tryptophan metabolism expression level, based on the absorbance value at 450nm to calculate the OD value.

### 2.9 SiRNA and adRNA transfection

Gene knockdown and overexpression fragment were carried out using the HiPerFect Transfection kit (Qiagen, Germany). AGS and SNU-1 cells were transfected with 5 nM si-ECHS1 and ad-ALDH2 following the protocol of manufacturer, and cells transfected with irrelevant non-targeting siRNA and adRNA, or were treated with sham transfection were used as negative control. The cells were cultured for another 24 h after transfection, and gene knockdown efficiency was measured by qRT-PCR.

### 2.10 Cell scratch

Seed AGS and SNU-1 cells onto the bottom of a six-well plate and mark scratch lines to create wounds. Remove the old culture medium and divide the cells into two groups: si-NC, ad-NC (negative control) and si-ECHS1 and ad-ALDH2. Place the plate in a 37°C, 5% CO2 cell culture incubator. At specified time points such as 0 hours and 48 hours, remove the cells from the plate and observe the width of the scratch at the same position under a microscope, taking pictures. Finally, use Image J software to analyze the distance of cell migration and the area of the scratch.

### 2.11 Statistical analysis

All data processing and statistical analysis were conducted using R software version 3.6.1 and GraphPad Prism. Student’s t-test and One-way analysis of variance (ANOVA) were used to determine differences between groups, and a p-value<0.05 indicated statistical significance.

## 3 Results

### 3.1 The expression of TMGs entirety landscape in GC

**Figure 1:**
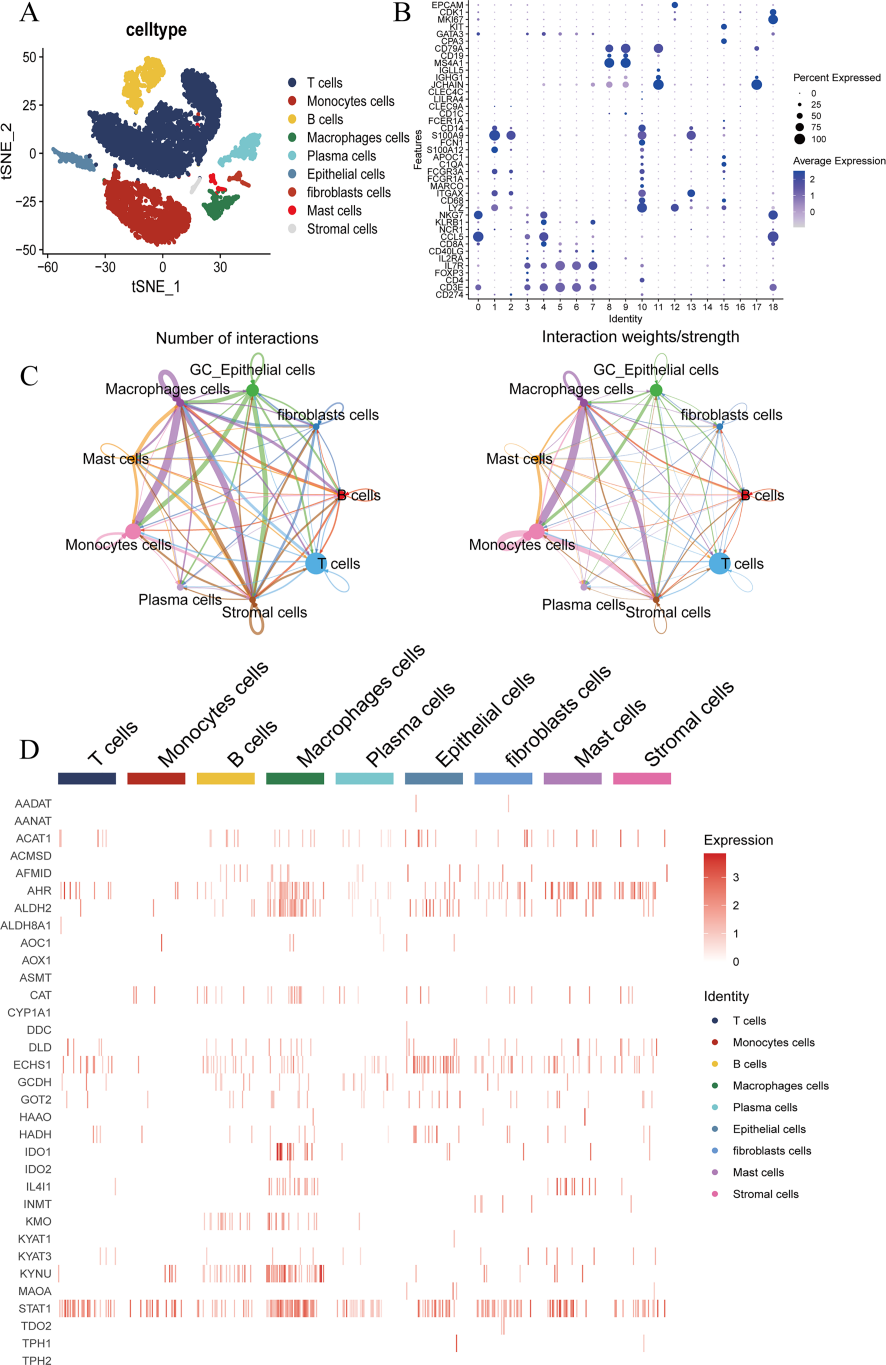
ScRNA-seq analysis of GC and normal groups. **(A, B)** Identification of TME cells type expression in GC including T cells, Monocytes cells, B cells, Macrophages cells, Plasma cells, Epithelial cells, Fibroblasts cells, Mast cells and Stromal cells in GSE163558. **(C)** The network diagram illustrates the interaction of key cell types in the GC. Each point represents a cell cluster, with the size of the point indicating the weight of that cluster in the network. The thickness of each line corresponds to its strength. **(D)** The expression of tryptophan metabolism family genes in key cell type of GC.

The expression patterns of TMGs have been observed across a spectrum of cancer types. To delve deeper into the differential expression of TMGs in GC, we utilized scRNA-seq data from GSE163558, encompassing 3 GC patients and 1 normal individual. This analysis facilitated the identification of nine distinct cell clusters within the TME based on markers. Including T cells, monocytes, B cells, macrophages, plasma cells, epithelial cells, fibroblasts, mast cells, and stromal cells (**Figures 1A, B**). The transformation of epithelial cells is a pivotal element in the progression of advanced GC, with a particular emphasis on tryptophan metabolism. After isolating tumor cells, we performed comprehensive cell communication analysis and identified a robust correlation between GC epithelial cells and macrophages. This observation suggests the presence of extensive cross-talk and interactions between macrophages and epithelial cells within the TME (**Figure 1C**). Subsequently, we conducted a comprehensive analysis of the gene expression profiles of TMGs, and observed that STAT1, ALDH2, and ECHS1 were highly expressed in GC epithelial cells (**Figure 1D**).

### 3.2 Heterogeneity of tumor microenvironment in GC

**Figure 2:**
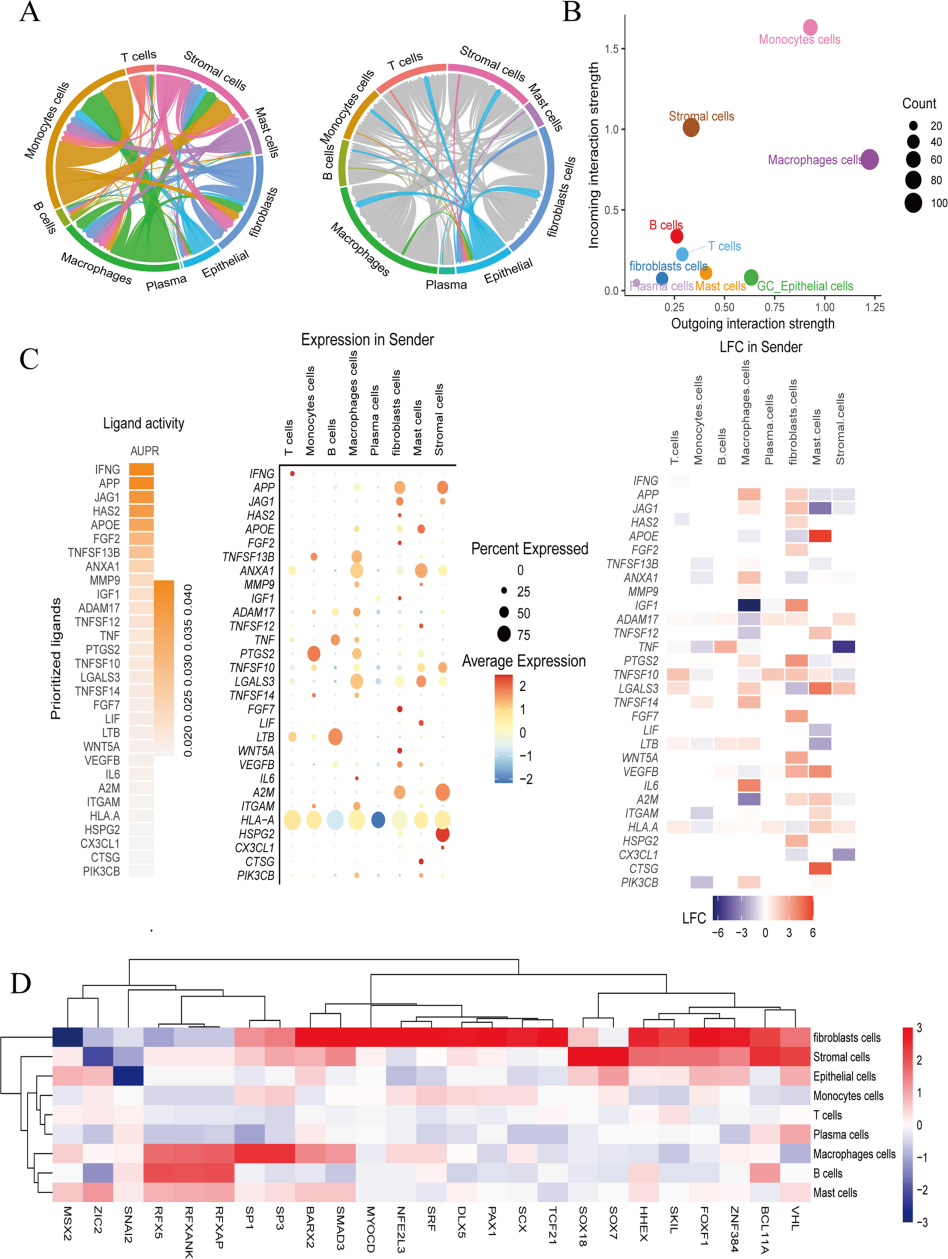
HLA-A ligands mediate a stronger tumor microenvironment regulated by FOXF1 and ZNF384. **(A, B)** The different expression of receptor-ligand in cell type. **(C)** The expression and function of HLA-A ligands in relation to the receptor on epithelial cells. Red indicates positive regulation, blue indicates negative regulation, and gray has no regulatory significance. **(D)** The expression of transcription factors varies among cell types. Red indicates positive regulation, blue indicates negative regulation, and gray has no regulatory significance.

To enhance our comprehension of the TME in GC, we discovered that each cell type manifests distinct receptor-ligand interactions, with epithelial cells notably prominent (**Figure 2A**). Epithelial cells are pivotal in dictating the intensity of outgoing interactions and are influenced by other cell types, which induces a shift from intracellular to extracellular functions. These interacting cell types encompass T cells, monocytes, B cells, macrophages, epithelial cells, fibroblasts, mast cells, and stromal cells (**Figure 2B**). Our analysis disclosed that HLA-A ligands, acting as crucial intermediaries, display differential expression patterns across various cell types within the TME, significantly impacting the regulation of epithelial cells (**Figure 2C**). Notably, the transcription factors FOXF1 and ZNF384 are implicated in the regulation of epithelial cell carcinogenesis at the protein transcription and translation levels (**Figure 2D**). Consequently, HLA-A ligands, through the modulation by FOXF1 and ZNF384, may facilitate the increased heterogeneity of the TME in GC.

### 3.3 Identification of key TMGs in GC

**Figure 3:**
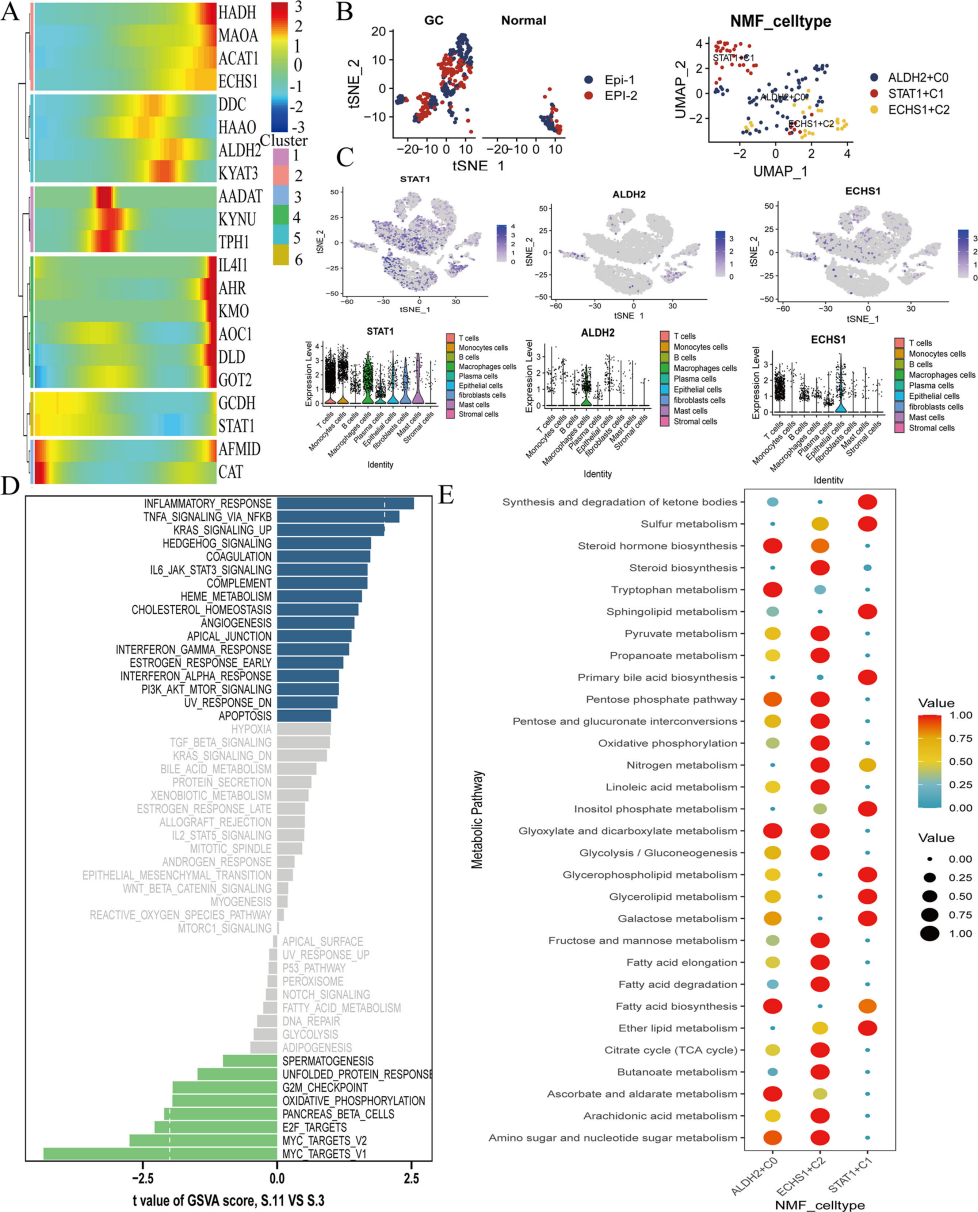
Identification of key tryptophan metabolism genes expression in GC. **(A)** The pseudo-time analysis the expression of TMGs-related genes, red indicates high expression and blue indicates low expression. **(B)**The process of extracting and visualizing epithelial cells through reclustering, which led to the identification of two distinct groups of epithelial cells, with epi-2 constituting the majority. The genes STAT1, ALDH2, and ECHS1 were specifically found to be expressed in the epi-2 group, as determined by NMF analysis. **(C)** The tSNE visualization displays the expression levels of STAT1, ALDH2, and ECHS1. **(D)** GSVA analysis highlights critical pathways in GC. **(E)**The tryptophan metabolism genes STAT1, ALDH2, and ECHS1 are significantly enriched in GC, with red indicating high expression levels.

Our analysis revealed that TMGs are prominently expressed during the middle and late stages of GC, specifically STAT1, ALDH2, and ECHS1 (**Figure 3A**). We identified two distinct types of epithelial cells, designated as Epi-1 and Epi-2. Subsequently, a deeper examination of Epi-2 cells indicated that three genes, STAT1, ALDH2, and ECHS1, could be discerned through Non-negative Matrix Factorization (NMF) analysis (**Figure 3B**). By tSNE visualization of TMGs-related genes,STAT1, ALDH2, and ECHS1, we found that it were widely expressed GC epithelial cell (**Figure 3C**). Furthermore, KEGG enrichment analysis underscored the NF-κB signaling pathway as critically important for the progression of GC (the blue color represents the positive correlation enrichment) (**Figure 3D**). Additionally, metabolic analysis observed that the TMGs, STAT1, ALDH2, and ECHS1, were significantly enriched in pathways such as the TCA cycle, Glyoxylate and dicarboxylate metabolism, Pentose phosphate pathway, and tryptophan metabolism (red means highly enriched). This implies that the TMGs, STAT1, ALDH2, and ECHS1, could influence the TCA cycle and contribute to the reprogramming of energy metabolism via the NF-κB signaling pathways (**Figure 3E**).

### 3.4 The differential expression of TMGs in GC transcriptome

**Figure 4:**
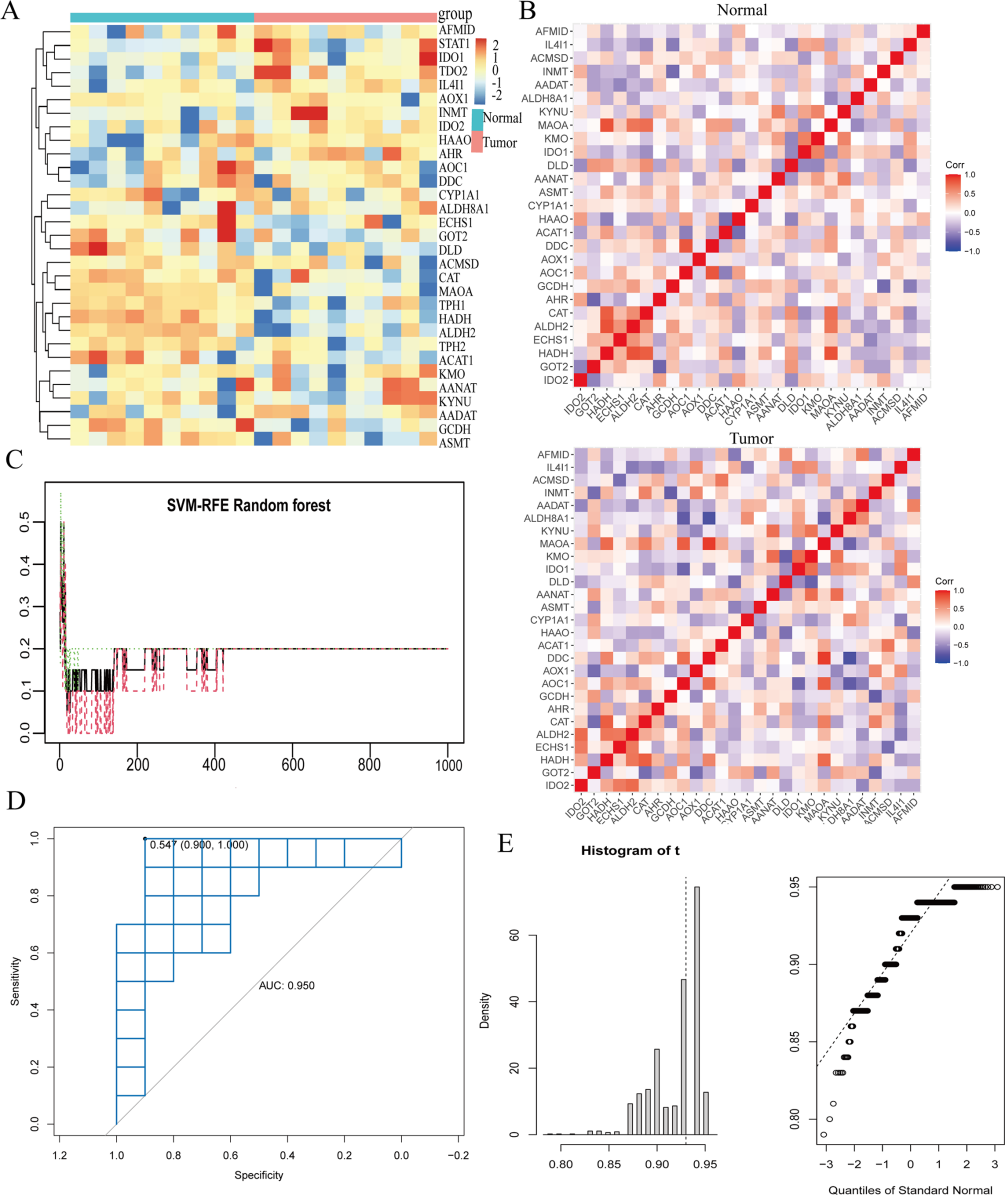
The SVM algorithm model was constructed for the tryptophan metabolism family genes. **(A)** The various expressions of tryptophan metabolism family genes in the GC transcriptome were analyzed. Red indicates high expression, blue indicates low expression. **(B)** The correlation of tryptophan metabolism family genes. The higher the correlation, the darker the color. **(C)** The SVM-RFE machine algorithm model was constructed in GSE79973. **(D, E)** ROC and calibration curve analysis of machine algorithm model predictive value in GSE79973. *, P<0.05,**, P<0.01, ***, P<0.001.

To corroborate our earlier findings, we examined the differential expression of TMGs ALDH2 and ECHS1 in the GC transcriptome, utilizing datasets from GSE79973 (**Figure 4A**). A subsequent correlation analysis between these genes in normal and GC tissues showed a robust correlation for the ECHS1 gene in tumor tissues, which was in stark contrast to the weak correlation observed in normal tissues. Conversely, the ALDH2 gene exhibited the opposite pattern (**Figure 4B**). We utilized the SVM-RFE technique to develop a machine learning algorithm model, evaluating its predictive accuracy. The model exhibited a high degree of accuracy in predicting GC, with an AUC of 0.950 at a minimum error value of 19 (**Figures 4C, D**). Furthermore, the calibration curve provided additional validation of the model’s precision (**Figure 4E**).

### 3.5 The ALDH2 and ECHS1 genes were promote the progression of GC

**Figure 5:**
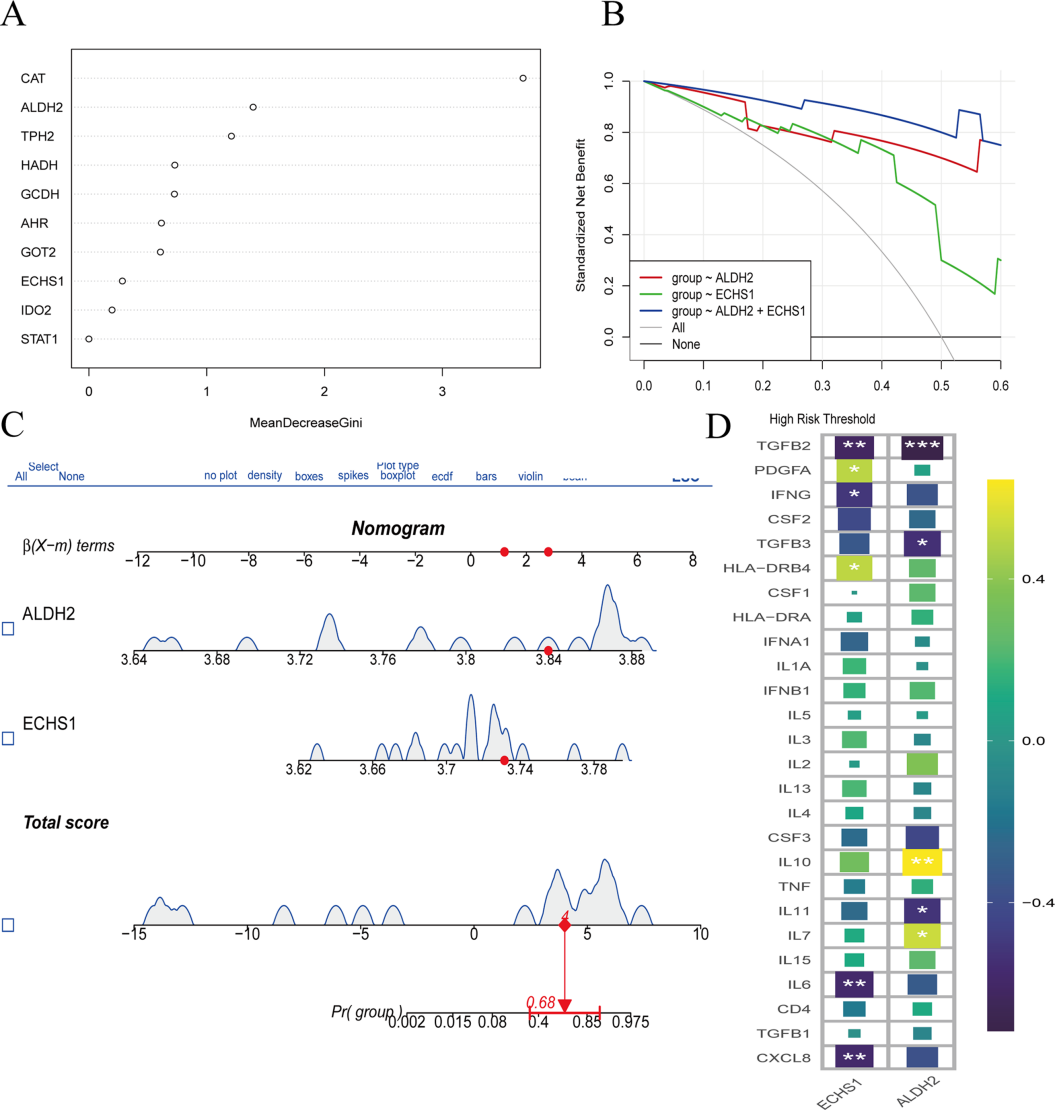
Transcriptomic analysis of tryptophan metabolic family genes expressed in GC and normal groups. The positive expression of tryptophan metabolism family genes were screened by svm algorithm. **(A)** RF model showed the top 10 tryptophan metabolic key genes in terms of importance. This graph is the MeanDecreaseGini coefficient graph, with MeanDecreaseGini values on the horizontal axis. The larger the MeanDecreaseGini value, the better the classification of categories. The vertical axis represents the expression of positive genes, arranged in descending order according to the MeanDecreaseGini coefficient. **(B, C)** Decision curve analysis (DCA) and nomogram predict the key gene expression in GC patients. **(D)** The correlation between the ALDH2 and ECHS1 genes and inflammation. *, P<0.05, **, P<0.01, ***, P<0.001.

Additionally, we discovered that the expression levels of ALDH2 and ECHS1 provide a more practical approach for the prognosis and prediction of outcomes in GC. We employed the Random Forest (RF) model to identify key genes involved in tryptophan metabolism that may influence the progression of GC (**Figure 5A**). Our findings also indicated that ALDH2 and ECHS1 serve as risk factors, with their expression being indicative of a poor prognosis for GC patients (**Figures 5B, C**). Furthermore, the ALDH2 and ECHS1 genes were associated with the relative aggregation of inflammatory factors, suggesting that the activation of tryptophan metabolism is linked to the release of inflammatory mediators, including IL6, IL7, IL10, CXCL8, TGFB3, TGFB2, IFNG, and PDGFA proteins (**Figure 5D**). Collectively, the involvement of ALDH2 and ECHS1 in tryptophan metabolism was found to promote the release of inflammatory factors, thereby contributing to a poorer prognosis.

### 3.6 ALDH2 and ECHS1 immunotherapy and prognosis by bulk transcriptome analysis in GC

**Figure 6:**
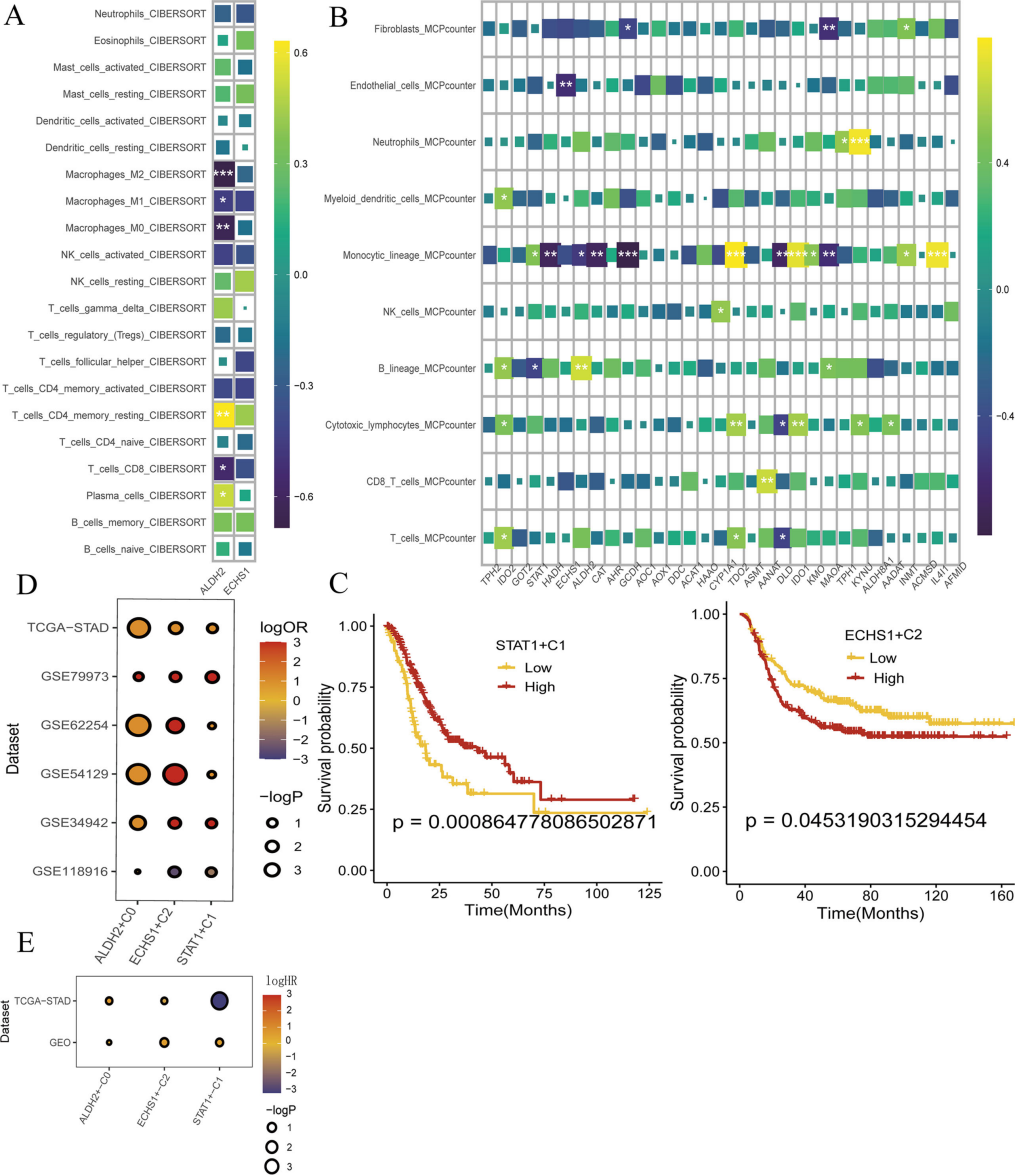
ALDH2 and ECHS1 ICB immunotherapy restrain the progress of GC. **(A, B)** Immune infiltration of ALDH2 and ECHS1 gene in GC. **(C)** To evaluate the key genes expression survival curves by Kaplan-Meier survival analysis. **(D)** Evaluation of the prognosis of ICB immunotherapy by TCGA-STAD, GSE79973, GSE62254, GSE54129, GSE34942 and GSE5118986. **(E)** bulk transcriptome analysis the expression of ALDH2 and ECHS1 and prognosis by TCGA-STAD, GSE54129 and GSE26253. *, P<0.05, **, P<0.01, ***, P<0.001.

We conducted an analysis to determine the correlation between ALDH2 and ECHS1 and the levels of various immune cells in GC. The findings revealed that ALDH2 was associated with disrupted immune levels, exhibiting a positive correlation with plasma cells, CD4+T cells, and NK cells, and a negative correlation with T-cells-CD8, Macrophages-M0, Macrophages-M1, and Macrophages-M2. In contrast, ECHS1 did not demonstrate any significant alterations in immune levels (**Figure 6A**). Furthermore, we employed the MCP counter to assess the immune infiltration score, which indicated that the expression of ALDH2 and ECHS1 influenced endothelial cell and monocyte cell function. Interestingly, these results aligned with our previous observations (**Figure 6B**). Subsequent analysis using bulk transcriptome data suggested that ALDH2 and ECHS1 may act as promoters of GC progression, while STAT1 could potentially function as a protective factor. Kaplan-Meier survival curves revealed that patients in the high-expression group of ECHS1 had significantly lower overall survival rates (p<0.05) (**Figures 6E, C**). Lastly, our findings indicated that the ALDH2 and ECHS1 proteins were associated with a better prognosis for GC when considering immunotherapy (ICB) (**Figure 6D**).

### 3.7 TMGs related genes affect the release of inflammatory cytokines in TME

**Figure 7:**
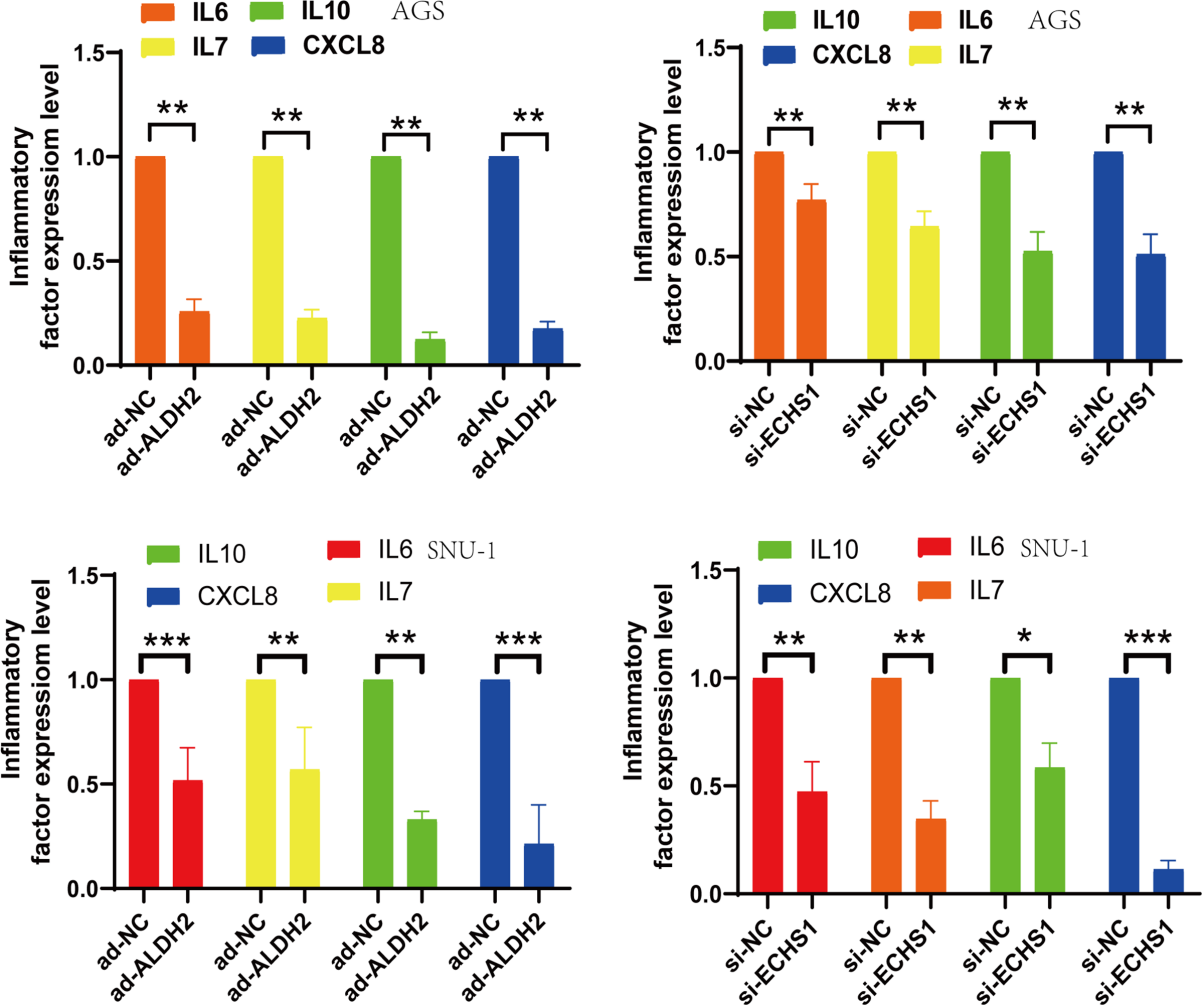
si-ECHS1 and ad-ALDH2 transfections were inhibition of inflammatory expression. The expression of IL6, IL7, IL10, CXCL8 by qPCR assay in AGS and SNU-1 cells. *, P<0.05, **, P<0.01, ***, P<0.001.

Chronic inflammation has always been an important factor in the complex TME.it stimulates vascular damage and epithelial tissue destruction. Our data demonstrated a decrease in the release of inflammatory mediators, including IL6, IL7, IL10, and CXCL8 (**Figure 7**).This also implies that inhibition of the expression of TMGs-related genes,ECHS1 and ALDH2, can regulate the expression of inflammatory factors and complex TME.

### 3.8 The down-regulated expression of ECHS1 and up-regulated expression ALDH2 decreased cell proliferation and migration in GC

**Figure 8:**
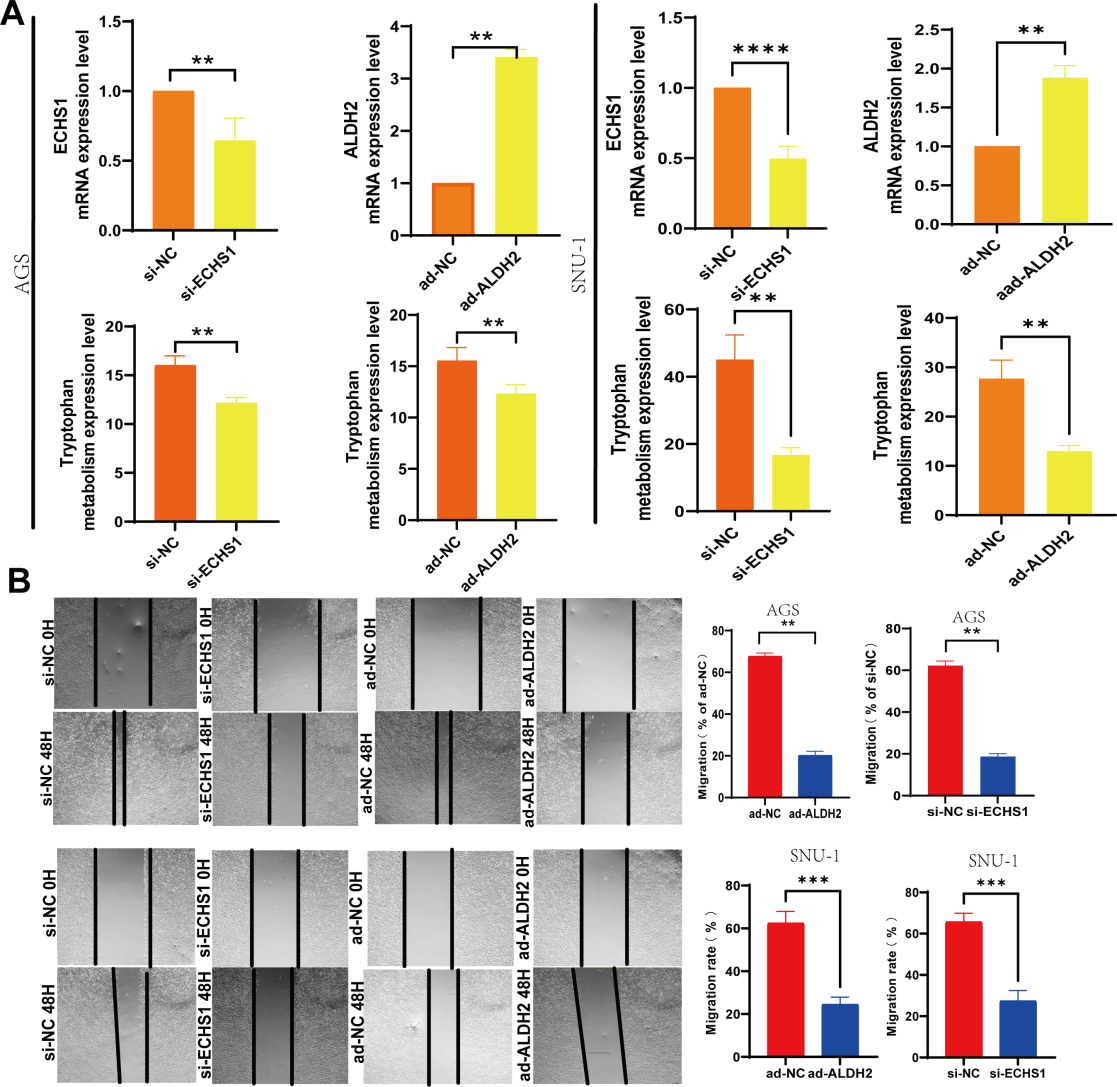
Low levels of tryptophan metabolism were effective in slowing the progression of GC. **(A)** The transfection of si-ECHS1 and ad-ALDH2 into AGS and SNU-1 cells influenced the expression of TMGs and tryptophan metabolism. **(B)** The influence of cell migration by the transfection of si-ECHS1 and ad-ALDH2 in AGS and SNU-1 cells. **, P<0.01, ***, P<0.001, ****, P<0.0001.

Moreover, through the application of interference and overexpression techniques in the transfection of GC cells, we noted a reduction in ECHS1 expression following si-ECHS1 and an enhancement in ALDH2 expression via ad-ALDH2. Concurrently, the metabolism of tryptophan was down-regulated as a result of transfection with si-ECHS1 and ad-ALDH2 (**Figures 8A, B**). Additionally, si-ECHS1 and ad-ALDH2 were inhibited the proliferation and migration of GC cells (**Figure 8B**).

## 4 Discussion

This research highlights the importance of ECHS1 and ALDH2 in GC progression and immune modulation, suggesting that targeted therapies focusing on these genes offer promising avenues for personalized immunotherapy in GC. These findings hold potential for improving patient survival and quality of life. Future studies on the NF-κB signaling pathway’s role in this context are warranted to further elucidate the mechanisms underlying TMG-mediated immune modulation in GC.

Recent studies have indicated that ALDH2 and ECHS1, as members of the TMGs, play a critical role in modulating immune responses in LUAD (29). It is worth noting that is famous for its cancer suppression characteristics of ALDH2 in LUAD and GC often lack of expression. This downregulation has been associated with increased cellular heterogeneity, leading to a more rapid tumor progression (25, 30). Furthermore, our findings reveal that the expression of ECHS1 is linked to various cancers, including colorectal cancer (31), hepatocellular carcinoma (32, 33), and breast cancer (34, 35), through its effects on metabolism and cell signaling pathways. These studies collectively suggest that the dysregulated expression of ALDH2 and ECHS1 can impair cellular functions, potentially contributing to the progression and worsening of gastric cancer.

In our current study, we have explored the correlation between TMGs (ALDH2 and ECHS1) and the progression of GC. Among these metabolites, ALDH2 and ECHS1 have been identified as particularly potent in preventing epithelial carcinogenesis. We observed an upregulation of ECHS1 and a downregulation of ALDH2 in GC cases. Notably, ALDH2 and ECHS1, derived from the clustering results in specific epithelial cells, were found to be significantly associated with molecular subtypes in tryptophan metabolism. Further analysis of various signaling pathways and immune-related attributes highlighted ALDH2 and ECHS1 as pivotal players in tryptophan metabolism, suggesting that the modulation of these genes could substantially influence the pathway’s regulation. Additionally, we developed a prognostic model that integrates both tryptophan metabolism and immune-related genes. Our data revealed that the expression of ECHS1 can lead to a poor prognosis in GC due to the impairment of CD8+ T cell and CD4+ T cell function. Moreover, ALDH2 and ECHS1 were found to regulate inflammation, including the expression of IL6, IL7, IL10, and CXCL8 proteins, and enhance TME heterogeneity through transcription factors ZNF384 and FOXF1, which is one of the significant causes of epithelial cell carcinogenesis in GC. Therefore, our data suggest that it can effectively predict the prognosis and immune therapy response of GC and guide personalized tryptophan metabolite-related targeted therapy.

Current research reports indicate that the NF-κB signaling pathways are pivotal in regulating both physiological and pathological processes. These pathways are implicated in responses to stimuli related to inflammation, immune response, and the heterogeneity of the TME (36). Several studies have demonstrated that the regulation of inflammation and natural aging is mediated by the NF-κB signaling pathways. Interestingly, our GSVA enrichment analysis revealed that the NF-κB signaling pathways specifically regulate TMGs, including IL6, IL7, IL10, and CXCL8 proteins (37, 38). Furthermore, we also discovered that the NF-κB signaling pathways are primarily involved in regulating and maintaining T cell function (39–41).

Our data indicate that the downregulated expression of ALDH2 contributes to a stronger immune invasion of CD8+ T cells and CD4+ T cells through the stimulation of NF-κB signaling pathways. However, ECHS1 did not exhibit changes in immune levels. Therefore, understanding the NF-κB signaling pathways is particularly crucial for immune therapy and maintaining T cell function. Nevertheless, there remains significant scope for additional research to fully comprehend the complexities involved. The potential of immune therapy in treating various diseases has garnered increasing interest, particularly for its capacity to target cancerous or aberrant cells by enhancing the body’s autoimmunity. Specifically, the study of ALDH2 and ECHS1 in immune therapy plays a crucial role in regulating GC. Additionally, the expression of ALDH2 and ECHS1 has a higher therapeutic value and can lead to improved survival outcomes through immune therapy. This can be achieved by utilizing samples from the TCGA and GEO databases. However, STAT1 appears to lack this therapeutic potential. In summary, ALDH2 and ECHS1, which are integral to tryptophan metabolism, serve as markers for prognostic prediction and viable targets for immunotherapy in GC patients.

It has been reported that chronic inflammation promotes the development of epithelial cancer, and the expression of ECHS1 and ALDH2 enhances the release of inflammatory factors, leading to a more robust TME. The release of inflammatory factors is likely associated with the NF-κB pathway. These findings suggest a dynamic cycle of chronic injury and repair, in which tryptophan metabolism plays a role. Importantly, interventions such as si-ECHS1 and ad-ALDH2 have been shown to significantly disrupt this dynamic cycle.

## 5 Conclusion

**Figure 9:**
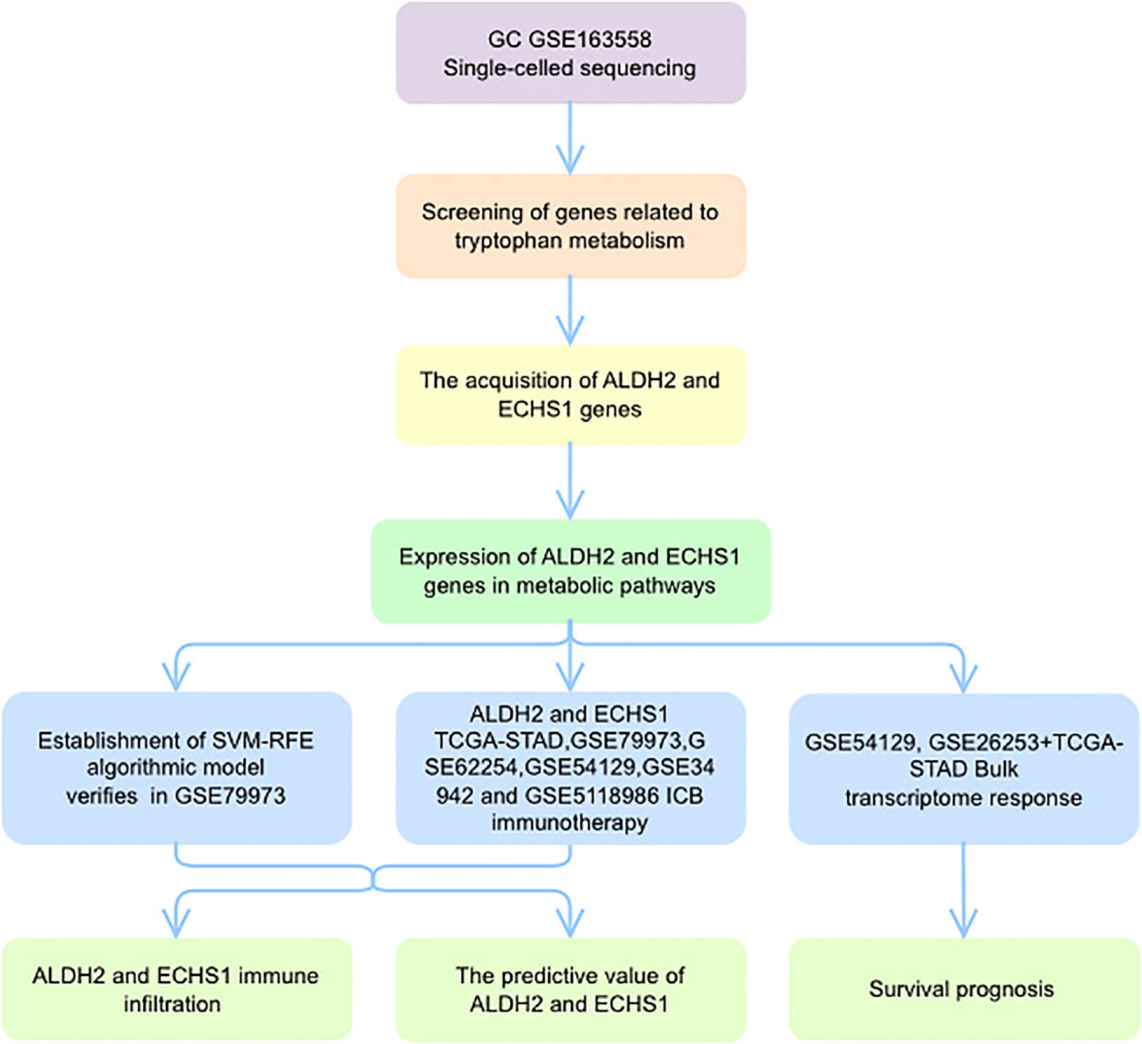
A global landscape of tryptophan metabolism analysis.

In conclusion, our study offers a new perspective for understanding the progression of the GC process (**Figure 9**). The TMGs- related genes ALDH2 and ECHS1 can contribute to TME heterogeneity and inflammation through the NF-κB signaling pathway. In addition, ALDH2 and ECHS1 may serve as novel tumor markers, providing a foundational basis for inhibiting GC progression. This study not only enhances our understanding of GC dynamics but also opens avenues for developing more effective therapeutic strategies.

## Data Availability

The original contributions presented in the study are included in the article/supplementary material. Further inquiries can be directed to the corresponding authors.

## References

[1] Luo P Chen G Shi Z Yang J Wang X Pan J. Comprehensive multi-omics analysis of tryptophan metabolism-related gene expression signature to predict prognosis in gastric cancer. Front Pharmacol. (2023) 14:1267186. doi: 10.3389/fphar.2023.1267186 37908977 PMC10613981

[2] Mahajan S Agashe D. Evolutionary jumps in bacterial GC content. G3 (Bethesda). (2022) 12. doi: 10.1093/g3journal/jkac108 PMC9339322 35579351

[3] Liu Y Liang N Xian Q Zhang W. GC heterogeneity reveals sequence-structures evolution of angiosperm ITS2. BMC Plant Biol. (2023) 23:608. doi: 10.1186/s12870-023-04634-9 38036992 PMC10691020

[4] Chen XJ Wei CZ Lin J Zhang RP Chen GM Li YF. Prognostic significance of PD-L1 expression in gastric cancer patients with peritoneal metastasis. Biomedicines. (2023) 11:1–12. doi: 10.3390/biomedicines11072003 PMC10377298 37509642

[5] Jin X Liu Z Yang D Yin K Chang X. Recent progress and future perspectives of immunotherapy in advanced gastric cancer. Front Immunol. (2022) 13:948647. doi: 10.3389/fimmu.2022.948647 35844558 PMC9284215

[6] Wang X Liu X Dai H Jia J. Peripheral blood nutrient indices as biomarkers for anti−PD−1 therapy efficacy and prognosis in patients with advanced gastric cancer. Oncol Lett. (2023) 26:397. doi: 10.3892/ol 37600335 PMC10433707

[7] Correia AS Vale N. Tryptophan metabolism in depression: A narrative review with a focus on serotonin and kynurenine pathways. Int J Mol Sci. (2022) 23:1–17. doi: 10.3390/ijms23158493 PMC9369076 35955633

[8] van Zundert SKM van Egmond NCM van Rossem L Willemsen SP Griffioen PH van Schaik RHN. First trimester maternal tryptophan metabolism and embryonic and fetal growth: the Rotterdam Periconceptional Cohort (Predict Study). Hum Reprod (Oxford England). (2024) 39:912–22. doi: 10.1093/humrep/deae046 PMC11063566 38498837

[9] Pirzadeh M Khalili N Rezaei N. The interplay between aryl hydrocarbon receptor, H. pylori, tryptophan, and arginine in the pathogenesis of gastric cancer. Int Rev Immunol. (2022) 41:299–312. doi: 10.1080/08830185.2020.1851371 33236682

[10] Li W Ling L Xiang L Ding P Yue W. Identification and validation of a risk model and molecular subtypes based on tryptophan metabolism-related genes to predict the clinical prognosis and tumor immune microenvironment in lower-grade glioma. Front Cell Neurosci. (2023) 17:1146686. doi: 10.3389/fncel.2023.1146686 36925967 PMC10011102

[11] Fu T Dai LJ Wu SY Xiao Y Ma D Jiang YZ. Spatial architecture of the immune microenvironment orchestrates tumor immunity and therapeutic response. J Hematol Oncol. (2021) 14:98. doi: 10.1186/s13045-021-01103-4 34172088 PMC8234625

[12] Ma G Zhang Z Li P Zhang Z Zeng M Liang Z. Reprogramming of glutamine metabolism and its impact on immune response in the tumor microenvironment. Cell Commun Signal. (2022) 20:114. doi: 10.1186/s12964-022-00909-0 35897036 PMC9327201

[13] Qin S Xu Y Yu S Han W Fan S Ai W. Molecular classification and tumor microenvironment characteristics in pheochromocytomas. eLife. (2024) 12. doi: 10.7554/eLife.87586 PMC10942623 38407266

[14] Li D Yu S Long Y Shi A Deng J Ma Y. Tryptophan metabolism: Mechanism-oriented therapy for neurological and psychiatric disorders. Front Immunol. (2022) 13:985378. doi: 10.3389/fimmu.2022.985378 36159806 PMC9496178

[15] Chen LM Bao CH Wu Y Liang SH Wang D Wu LY. Tryptophan-kynurenine metabolism: a link between the gut and brain for depression in inflammatory bowel disease. J Neuroinflamm. (2021) 18:135. doi: 10.1186/s12974-021-02175-2 PMC8204445 34127024

[16] Michaudel C Danne C Agus A Magniez A Aucouturier A Spatz M. Rewiring the altered tryptophan metabolism as a novel therapeutic strategy in inflammatory bowel diseases. Gut. (2023) 72:1296–307. doi: 10.1136/gutjnl-2022-327337 PMC10314090 36270778

[17] Ilie-Mihai RM Stefan-van Staden RI Magerusan L Coros M Pruneanu S. Enantioanalysis of tryptophan in whole blood samples using stochastic sensors-A screening test for gastric cancer. Chirality. (2020) 32:215–22. doi: 10.1002/chir.23155 31747471

[18] Yao S Yin X Chen T Chen W Zuo H Bi Z. ALDH2 is a prognostic biomarker and related with immune infiltrates in HCC. Am J Cancer Res. (2021) 11:5319–37. PMC8640816 34873463

[19] Chi H Chen H Wang R Zhang J Jiang L Zhang S. Proposing new early detection indicators for pancreatic cancer: Combining machine learning and neural networks for serum miRNA-based diagnostic model. Front Oncol. (2023) 13:1244578. doi: 10.3389/fonc.2023.1244578 37601672 PMC10437932

[20] Yao Q Zhang X Wang Y Wang C Wei C Chen J. Comprehensive analysis of a tryptophan metabolism-related model in the prognostic prediction and immune status for clear cell renal carcinoma. Eur J Med Res. (2024) 29:22. doi: 10.1186/s40001-023-01619-0 38183155 PMC10768089

[21] Li P Xu W Liu F Zhu H Zhang L Ding Z. The emerging roles of IDO2 in cancer and its potential as a therapeutic target. BioMed Pharmacother. (2021) 137:111295. doi: 10.1016/j.biopha.2021.111295 33550042

[22] Hu T Chen X Lu S Zeng H Guo L Han Y. Biological role and mechanism of lipid metabolism reprogramming related gene ECHS1 in cancer. Technol Cancer Res Treat. (2022) 21. doi: 10.1177/15330338221140655 PMC9806408 36567598

[23] Chi H Huang J Yan Y Jiang C Zhang S Chen H. Unraveling the role of disulfidptosis-related LncRNAs in colon cancer: a prognostic indicator for immunotherapy response, chemotherapy sensitivity, and insights into cell death mechanisms. Front Mol Biosci. (2023) 10:1254232. doi: 10.3389/fmolb.2023.1254232 37916187 PMC10617599

[24] Zhang S Jiang C Jiang L Chen H Huang J Zhang J. Uncovering the immune microenvironment and molecular subtypes of hepatitis B-related liver cirrhosis and developing stable a diagnostic differential model by machine learning and artificial neural networks. Front Mol Biosci. (2023) 10:1275897. doi: 10.3389/fmolb.2023.1275897 37808522 PMC10556489

[25] Lu T Sun L Fan Q Yan J Zhao D Xu C. Expression and clinical significance of ECHS1 in gastric cancer. J Cancer. (2024) 15:418–27. doi: 10.7150/jca.88604 PMC10758025 38169583

[26] Zhang S Jiang C Jiang L Chen H Huang J Gao X. Construction of a diagnostic model for hepatitis B-related hepatocellular carcinoma using machine learning and artificial neural networks and revealing the correlation by immunoassay. Tumour Virus Res. (2023) 16:200271. doi: 10.1016/j.tvr.2023.200271 37774952 PMC10638043

[27] Khan SU Huang Y Ali H Ali I Ahmad S Khan SU. Single-cell RNA sequencing (scRNA-seq): advances and challenges for cardiovascular diseases (CVDs). Curr Probl Cardiol. (2024) 49:102202. doi: 10.1016/j.cpcardiol.2023.102202 37967800

[28] Takahashi H Hisata K Iguchi R Kikuchi S Ogasawara M Satoh N. scRNA-seq analysis of cells comprising the amphioxus notochord. Dev Biol. (2024) 508:24–37. doi: 10.1016/j.ydbio.2024.01.003 38224933

[29] Tran TO Vo TH Lam LHT Le NQK. ALDH2 as a potential stem cell-related biomarker in lung adenocarcinoma: Comprehensive multi-omics analysis. Comput Struct Biotechnol J. (2023) 21:1921–9. doi: 10.1016/j.csbj.2023.02.045 PMC10018390 36936815

[30] Yao S Gan C Wang T Zhang Q Zhang M Cheng H. High ALDH2 expression is associated with better prognosis in patients with gastric cancer. Am J Cancer Res. (2022) 12:5425–39. PMC9827082 36628272

[31] Li R Hao Y Wang Q Meng Y Wu K Liu C. ECHS1, an interacting protein of LASP1, induces sphingolipid-metabolism imbalance to promote colorectal cancer progression by regulating ceramide glycosylation. Cell Death Dis. (2021) 12:911. doi: 10.1038/s41419-021-04213-6 34615856 PMC8494735

[32] Shihana F Cholan PM Fraser S Oehlers SH Seth D. Investigating the role of lipid genes in liver disease using fatty liver models of alcohol and high fat in zebrafish (Danio rerio). Liver International: Off J Int Assoc Study Liver. (2023) 43:2455–68. doi: 10.1111/liv.15716 37650211

[33] Wang L Qi Y Wang X Li L Ma Y Zheng J. ECHS1 suppresses renal cell carcinoma development through inhibiting mTOR signaling activation. Biomed Pharmacother. (2020) 123:109750. doi: 10.1016/j.biopha.2019.109750 31891870

[34] Shi Y Qiu M Wu Y Hai L. MiR-548-3p functions as an anti-oncogenic regulator in breast cancer. BioMed Pharmacother. (2015) 75:111–6. doi: 10.1016/j.biopha.2015.07.027 26297544

[35] Muntean C Tripon F Bogliş A Bănescu C. Pathogenic biallelic mutations in ECHS1 in a case with short-chain enoyl-coA hydratase (SCEH) deficiency-case report and literature review. Int J Environ Res Public Health. (2022) 19:1–15. doi: 10.3390/ijerph19042088 PMC8871535 35206276

[36] Yu H Lin L Zhang Z Zhang H Hu H. Targeting NF-κB pathway for the therapy of diseases: mechanism and clinical study. Signal Transduct Target Ther. (2020) 5:209. doi: 10.1038/s41392-020-00312-6 32958760 PMC7506548

[37] Shelby A Pendleton C Thayer E Johnson GK Xie XJ Brogden KA. PD-L1 correlates with chemokines and cytokines in gingival crevicular fluid from healthy and diseased sites in subjects with periodontitis. BMC Res Notes. (2020) 13:532. doi: 10.1186/s13104-020-05376-9 33187554 PMC7666489

[38] Xue L Wang C Qian Y Zhu W Liu L Yang X. Tryptophan metabolism regulates inflammatory macrophage polarization as a predictive factor for breast cancer immunotherapy. Int Immunopharmacol. (2023) 125:111196. doi: 10.1016/j.intimp.2023.111196 37972471

[39] Daniels MA Luera D Teixeiro E. NFκB signaling in T cell memory. Front Immunol. (2023) 14:1129191. doi: 10.3389/fimmu.2023.1129191 36911729 PMC9998984

[40] Rui R Zhou L He S. Cancer immunotherapies: advances and bottlenecks. Front Immunol. (2023) 14:1212476. doi: 10.3389/fimmu.2023.1212476 37691932 PMC10484345

[41] Chi H Gao X Xia Z Yu W Yin X Pan Y. FAM family gene prediction model reveals heterogeneity, stemness and immune microenvironment of UCEC. Front Mol Biosci. (2023) 10:1200335. doi: 10.3389/fmolb.2023.1200335 37275958 PMC10235772

